# Cellularizing hydrogel-based scaffolds to repair bone tissue: How to create a physiologically relevant micro-environment?

**DOI:** 10.1177/2041731417712073

**Published:** 2017-06-08

**Authors:** Mathieu Maisani, Daniele Pezzoli, Olivier Chassande, Diego Mantovani

**Affiliations:** 1 Laboratory for Biomaterials & Bioengineering (CRC-I), Department Min-Met-Materials Engineering & Research Center CHU de Québec, Laval University, Québec City, QC, Canada; 2 Laboratoire BioTis, Inserm U1026, Université de Bordeaux, Bordeaux, France

**Keywords:** Stem cells, hydrogels, bone tissue engineering, micro-environment

## Abstract

Tissue engineering is a promising alternative to autografts or allografts for the regeneration of large bone defects. Cell-free biomaterials with different degrees of sophistication can be used for several therapeutic indications, to stimulate bone repair by the host tissue. However, when osteoprogenitors are not available in the damaged tissue, exogenous cells with an osteoblast differentiation potential must be provided. These cells should have the capacity to colonize the defect and to participate in the building of new bone tissue. To achieve this goal, cells must survive, remain in the defect site, eventually proliferate, and differentiate into mature osteoblasts. A critical issue for these engrafted cells is to be fed by oxygen and nutrients: the transient absence of a vascular network upon implantation is a major challenge for cells to survive in the site of implantation, and different strategies can be followed to promote cell survival under poor oxygen and nutrient supply and to promote rapid vascularization of the defect area. These strategies involve the use of scaffolds designed to create the appropriate micro-environment for cells to survive, proliferate, and differentiate in vitro and in vivo. Hydrogels are an eclectic class of materials that can be easily cellularized and provide effective, minimally invasive approaches to fill bone defects and favor bone tissue regeneration. Furthermore, by playing on their composition and processing, it is possible to obtain biocompatible systems with adequate chemical, biological, and mechanical properties. However, only a good combination of scaffold and cells, possibly with the aid of incorporated growth factors, can lead to successful results in bone regeneration. This review presents the strategies used to design cellularized hydrogel-based systems for bone regeneration, identifying the key parameters of the many different micro-environments created within hydrogels.

## Introduction

Severe bone lesions cause hundreds of millions of surgical procedures each year around the world. Bone is a dynamic and vascularized tissue that has the ability of naturally healing upon damage. Nevertheless, in the case of large defects (such as in non-union fractures,^1^ maxillofacial trauma,^2,3^ tumor ablations,^4,5^ intervertebral disk injury or degeneration^6,7^), this potential is impaired and surgical procedures including the use of autografts, allografts, or grafting of exogenous biomaterials are necessary. These grafted materials must ensure mechanical stability and provide the appropriate environment for efficient healing.^8,9^ These approaches present several limitations: (1) autografts may involve tissue morbidity, and moreover, the availability of donor tissue is limited; (2) allografts cause an important risk of infection and immunogenic rejection mechanisms; and (3) solid biomaterials such as metal or ceramic implants do not easily fit the size and shape of the defect.^10^ Although recent advances in three-dimensional (3D) printing of solid materials have enabled the fabrication of size and shape-controlled materials, their surgical implantation to fit the morphology of the damaged site is far from easy. In this context, new classes of biomaterials for bone healing are the focus of much research. A promising strategy for the regeneration of bone is bone tissue engineering (BTE), based on the use of 3D matrices (scaffolds) to guide cellular growth and differentiation and to promote the deposition of new bone tissue.^11^ Hydrogels are among the most promising biomaterials in BTE applications since they are very flexible materials that allow several different properties to be targeted for specific applications and they can be formulated to be implantable with minimal invasive procedures. In fact, ideally hydrogels should be injectable. In contrast to rigid scaffolds, hydrogels can establish tight contacts with the host tissue, limiting fibrosis and favoring osteoconductivity. The only limitation of hydrogels is their low stiffness, which does not allow their use for the repair of load-bearing lesions, such as large fractures of long bones. Instead, hydrogels rather appear as lesion filling materials. Hydrogels are hydrophilic polymeric 3D networks which can contain and/or release in a controlled fashion cells for tissue regeneration and/or bioactive molecules such as growth factors.^8^ The cells encapsulated in hydrogel systems can exert two types of effects. They can directly take part as building blocks in tissue regeneration, and in such case their long-term survival is required. Alternatively, they can stimulate host responses, ultimately favoring tissue repair.^12^ In this latter case, transient persistence of these cells may be sufficient. Whatever the mechanisms, the choice of the appropriate progenitor cells and of appropriate culture conditions prior to incorporation in the hydrogel scaffold is the key issue for the efficiency of BTE products.

This review, after describing the physiology of bone tissue and its healing mechanisms, is intended to provide a critical overview of the cells employed for bone tissue regeneration and of hydrogel-based scaffolds as optimal, potentially injectable, physiologically relevant micro-environments for the survival, recruitment, proliferation, and differentiation of bone cells in BTE applications. Relevant examples in the literature will be examined attempting to determine the key parameters which may influence cell behavior and fate, at each of the many different steps of the preparation of tissue engineering hydrogel-based constructs.

## Bone physiology and healing mechanisms

### Bone structure

Bone is a connective tissue that can be considered as a composite cellularized living material typically composed of an inner spongy bone, often named cancellous bone or trabecular bone, and an outer compact bone also defined as cortical bone, whose relative mass ratio is 20%–80% in the whole skeletal system.^13^

Cortical bone is composed of osteons, or haversian systems, cylindrical structures around 200 µm in diameter, with an inner channel (the haversian canal) containing blood vessels surrounded by concentric lamellae of mineralized matrix among which small cavities called lacunae are present, each containing an osteocyte. A network of small channels (canaliculi) connect the lacunae and the haversian canals, allowing cell–cell interactions and favoring exchange of nutrients and metabolites.

The honeycomb-like network of trabeculae forming cancellous bone also has a lamellar organization, but internal canals and blood vessels are missing. The trabecular network in fact is filled with marrow, a tissue composed of blood vessels, nerves, and several cell types, from which trabecular osteocytes receive nutrients.

Bone extracellular matrix (ECM) is characterized by two phases: an inorganic mineral component and an organic protein phase. The inorganic component provides stiffness to the bone and is mainly composed of hydroxyapatite (HA; Ca_10_(PO_4_)_6_(OH)_2_) crystals, even if calcium carbonate, calcium fluoride, and magnesium fluoride are also present, and serves as reservoir for the homeostasis of ions, containing 99% of calcium and 88% of phosphate of the human body.^14^

The organic component is mainly composed of a network of type I collagen triple helices organized in fibrils (ca. 90%), the remaining part being non-collagenous proteins such as glycoproteins, proteoglycans (PGs), and growth factors. The structural role of the organic ECM is twofold: regulating the nucleation and direction of HA crystals and thus the shape of the bone and providing ductility and fracture toughness. In addition, the inorganic ECM is a reservoir for growth factors and cytokines involved in bone remodeling and reparation.

Among non-collagenous proteins, approximately 10% is constituted of PGs, macromolecules composed of strongly hydrophilic negatively charged long carbohydrate chains (glycosaminoglycans (GAGs), mainly chondroitin sulfate, dermatan sulfate, keratan sulfate, and hyaluronic acid) covalently linked to a core protein.^15^ PGs form a highly hydrated swelled gel-like matrix whose main role is to provide resistance to compressive stress. In addition, PGs function as growth factors’ binding and storage agents and as regulators of collagen fibrillogenesis.^16^ Bone ECM glycoproteins include alkaline phosphatase (ALP), osteopontin, bone sialoprotein, and osteocalcin, all involved in the mineralization process.

### Bone remodeling

Bone growth, modeling, and remodeling are lifelong processes meant to guarantee tissue size and shape adaptation, structural integrity, and regulation of mineral homeostasis. Bone growth occurs mainly during childhood and adolescence; modeling consists of the gradual transformation of bone shape in response to the applied mechanical forces; finally, bone remodeling is the continuous process by which bone tissue is renewed to maintain its integrity and strength and to control mineral homeostasis.

Bone remodeling is tightly regulated by the orchestral action of an ensemble of multiple cell types arranged within temporary bone remodeling compartments known as basic multicellular units or bone metabolic units (BMUs).^17^ Remodeling is initiated by remodeling signals (activation phase) that can be hormones such as parathyroid hormone (PTH), secreted to maintain calcium homeostasis, or mechanical stimuli detected by osteocytes, inactive osteoblasts with low metabolic function located in bone lacunae that serve as stress and strain sensors and express paracrine signals for active osteoblasts and osteoclasts (e.g. the inhibitory osteoclastogenesis signal transforming growth factor-β (TGF-β)), thus directing bone turnover. However, it is osteoblasts that in response to remodeling signals can produce osteoclastogenesis cytokines and directly recruit osteoclast precursors and promote their proliferation and differentiation into multinucleated osteoclasts that begin the bone resorption phase. Osteoclasts derive from mononuclear precursors of the hematopoietic lineage that upon stimulation by cytokines produced by osteoblasts undergo fusion forming large multinucleated cells.^18^ Bone resorption is achieved by secretion of H^+^ through membrane proton pumps that create an acidic environment, with a pH as low as 4.5, in the resorptive pit (Howship lacuna) that dissolves the mineral component of the matrix while kathepsin K and other enzymes are released to break down the organic ECM. Then osteoclasts undergo apoptosis and are substituted by mononuclear cells of still unclear phenotype (reversal cells) that conclude the resorption phase and prepare the surface for the deposition of new matrix.^19^ The resorptive pit is then occupied by the osteocytes released from the resorbed matrix, Mesenchymal Stromal cells (MSCs), and preosteoblasts that are recruited from the medullary cavity or the periosteum, a fibrous membrane covering the external surface of bones populated by progenitor cells, by mature osteoclasts, reversal cells, and signals liberated from the degraded bone matrix. Mature osteoblasts are generated by differentiation of progenitor cells by growth factors such as bone morphogenetic proteins (BMPs), fibroblast growth factor (FGF), and TGF-β and are then responsible for the synthesis of new bone ECM. As osteoblasts end depositing new matrix, three main possible fates are possible for them: (1) remaining embedded in the newly formed mineralized tissue and transforming into osteocytes, (2) undergoing apoptosis, or (3) becoming bone lining cells, quiescent osteoblasts with a flat morphology that populate non-remodeling bone surfaces.^20^

A comprehensive description of the cellular and molecular mechanisms coordinating the different phases of bone remodeling is beyond the purposes of this review and it has been extensively reviewed elsewhere.^17,19^

### Bone healing

Bone has an intrinsic ability to repair itself. Bone healing processes are not fully understood, but their understanding is the key to the design and development of new effective strategies for the treatment of non-healing bone defects. When fractures occur, locally, the skeletal integrity is lost and the bone vascular network is disrupted leading to impaired nutrient and oxygen flow and affecting the marrow structure.^21^ Then the tissue regeneration process begins following three main phases: the inflammation (reactive) phase, the reparative phase, and the remodeling phase.^21^

In the early inflammation phase, a blood clot (hematoma) is locally formed, and growth factors (e.g. insulin-like growth factor I (IGF-I) and platelet-derived growth factor (PDGF)) and cytokines are released to attract and regulate the action of monocyte–macrophages and osteo-chondroblast precursor cells. Then the recruited immune cells secrete signaling molecules (e.g. FGF, tumor necrosis factor-α (TNF-α), vascular endothelial growth factor (v-EGF), TGF-β, interleukin-1 and interleukin-6 (IL-1, IL-6)) that stimulate ECM synthesis and angiogenesis and chemotactically attract other inflammatory cells and mesenchymal cell precursors (mainly originating from the periosteum) that proliferate and differentiate into chondrogenic and osteogenic lineages,^22^ finally forming a transient granulation tissue.

In the reparative phase, the so-called fracture callus is generated by one of the two following ossification processes: endochondral ossification and intramembranous ossification. In endochondral ossification, chondroblasts deposit a cartilaginous callus bridging and stabilizing the fracture site that is then calcified, vascularized, and gradually substituted by osteoblasts with woven bone, mechanically weak, and characterized by a random organization of the collagen fibers. In intramembranous ossification, both compact and trabecular bone are directly synthesized by osteoblasts without the intermediary cartilage deposition phase. This process is mainly limited to the subperiostal regions adjacent to both the ends of the fracture^23^ and the bone marrow, characterized by a functional capillary network and high O_2_ tension.^22^

Finally, in the remodeling phase, the fracture callus is converted into new bone tissue with a lamellar structure and an inner medullary cavity, thus finally fully restoring the biomechanical properties of the bone.^23^ Similarly to bone remodeling in intact bones, the remodeling phase of the bone healing process is based on BMUs and consists of a combination of callus resorption by osteoclasts and bone deposition by osteoblasts and may take years to achieve the fully repaired bone.

For a complete description of the biology of fracture healing, involving the tight coordination of several cell types and changes in the expression of thousand genes, the reader is referred to specific comprehensive reviews.^22,23^

#### MSCs in bone healing

MSCs play a pivotal role in bone healing by differentiating into chondroblasts and osteoblasts that deposit the fracture callus in the reparative phase.^24^ They are mainly recruited from the bone marrow and the periosteum, even if a systemic recruitment of MSCs circulating in the blood is also possible.^25^ The process regulating MSC recruitment in the site of injury is still not completely understood, since it is often difficult to clearly discriminate effects on recruitment, proliferation, and differentiation. However, it is generally agreed that they migrate along chemical gradients of potent chemokines and growth factors by chemotaxis, and stromal cell–derived factor-1 (SDF-1) is currently the most recognized recruitment signal.^26^

Also, the molecular mechanisms governing proliferation and differentiation of MSCs are still not fully elucidated. Several signaling pathways are involved in parallel, encompassing FGF, BMPs, Wnt, and Notch signaling, and also physiologic stimuli such as mechanical strain and hypoxia.^24^ The combination of these differentiation signals, during bone healing, can lead to the production of osteoblasts or chondrocytes that finally accomplish bone formation. In addition, MSCs can play an indirect trophic role in fracture healing by secreting cytokines and growth factors thus contributing in the recruitment of other cells, in the stimulation of vascularization and in the modulation of immunological responses.^24,27^

## Hydrogels: suitable micro-environments for BTE

Some large fractures or lesions caused by loss of large amounts of trabecular bone cannot self-heal and require biomaterials either as substitute or as filler to restore the mechanical properties of the damaged organ.^28^ In these situations, regeneration of damaged bone necessitates either an osteoconductive biomaterial, which will enable good osseointegration, or an osteoinductive system, which will enable the recruitment and differentiation of host cells. In some instances, osteoblast progenitors are not available in the vicinity of the lesion, and exogenous stem cells may be implanted. This therapeutic approach is known as tissue engineering, in which a scaffold is associated with stem cells and growth factors to be implanted in severe lesions, to promote efficient formation of new vascularized bone, with biological and mechanical characteristics as close as possible to those of native bone.

Biomaterials for bone repair must be able to provide temporary structural and mechanical support to the tissue regenerating cells which will colonize them, allowing their proliferation, possibly the differentiation in suitable cell types and finally the synthesis of a mineralized bone matrix that will replace the scaffold itself. As described in the previous section, the bone micro-environment is complex, and consequently several properties are required for the 3D scaffold material to favor an adequate regeneration of the bone tissue.^29^

First, a scaffold material must be biocompatible, generally meaning that upon implantation it must not cause an important deleterious inflammatory reaction or other adverse topic or systemic effects and should not be toxic for the recipient tissues and the cells it can harbor.^30,31^ The material should be bioactive, particularly at the interface with the host tissue allowing (1) the establishment of bonds and connections with the surrounding bone and thus a rapid osseointegration and (2) the colonization of the scaffold by osteoprogenitor and differentiated bone cells that can promote the deposition of new bone tissue. In this context, scaffolds for bone regeneration should satisfy three main properties: osteoconductivity, osteoinductivity, and osteogenicity.

Osteoconductivity is the ability of the material to favor bone growth at the biomaterial–host interface that is on the external and internal surfaces of the scaffold. Osteoconduction is perpetrated by stimulating the adhesion and migration of cells from the surrounding bone within the material and the deposition of new bone tissue.^32^ It is dependent on the physical, chemical, and structural (e.g. porosity) properties of the scaffold, and also mechanical properties, biocompatibility, biodegradability, and hydrophilicity will influence it.^8^

Osteoinductivity is the bioactive ability of the scaffold to recruit stem cells and promote their differentiation toward osteogenic lineages, thus inducing bone regeneration. Osteoinduction can be stimulated by material chemical properties, by its structure (macrostructure, microstructure and nanostructure), or by the presence of osteoinductive growth factors such as BMPs.^33,34^

Osteogenicity implies the presence of osteoprogenitor cells inside the graft (e.g. autografts) or the scaffold material and their proliferation to create a cellular environment prone to osteogenesis.^35^

Hydrogels are three-dimensional strongly hydrophilic polymer networks which can absorb huge quantities of water and that mimic the characteristics of the ECM of native tissues, providing cells with a temporary mechanical support while guaranteeing adequate nutrient and gas exchange.^36^ This provides an ideal micro-environment for cellular proliferation and differentiation, thus allowing bone cells encapsulated/migrating in the hydrogel to grow and secrete new ECM for restoration of damaged bone tissue.^37^ Thanks to all these advantages, hydrogels are increasingly considered as the option of choice for bone regeneration. In addition, hydrogels can possibly be loaded with bioactive molecules, osteoconductive/osteogenic growth factors, or with cells and injected in the site of morbidity before gelation.^38^ These injectable hydrogels permit less invasive surgical procedures, with respect to hard scaffolds, regardless of the shape of the bone lesion since they can also easily fill irregularly shaped defects.^39^

Owing to the plethora of advantages offered by hydrogels, this section will focus on the methods of preparation, the properties, and the composition of hydrogels as systems providing a physiologically relevant environment for cell adhesion, proliferation, and differentiation for BTE strategies.

### Methods of preparation of hydrogels

The mechanical (visco)elastic behavior and the extent of swelling of hydrogels depend on the balance between the osmotic forces that promote water inflow and the cohesive forces that resist the deformation of the polymeric 3D network and impart it mechanical reinforcement and stress resistance. Therefore, the swelling ratio strongly depends on the chemical properties of the polymeric components (i.e. hydrophilicity) and on the type and extent of crosslinking.^40^ It is thus clear that by playing with the method of preparation and with the parameters of the crosslinking reaction, it is possible to tune the final properties of hydrogels.

For the preparation of hydrogels, hydrophilic polymers are crosslinked either through covalent bonds or via physical intra- and intermolecular interactions. The main methods of preparation of hydrogels together with their main advantages and limitations are summarized in Table 1.

**Table 1.** Methods of preparation of hydrogels.

Type of crosslinking	Example of material	Advantages	Limitations	Ref.
Ionic crosslinking (physical)	Alginate Pectin	Cytocompatibility Cell encapsulation/injectability	Poor mechanical properties Low stability in physiological environments	6, 37, 41
Hydrogen bonding (physical)	Gelatin	Cytocompatibility Cell encapsulation/injectability	Poor mechanical properties Low stability in physiological environments	42–44
Hydrophobic association (physical)	ESHU PNIPAAm Pluronic^®^	Thermoresponsiveness Cytocompatibility LCST tunable at ~37°C Cell encapsulation/injectability	Poor mechanical properties	37, 40, 43, 45
Free radical polymerization (covalent)	Vinyl monomer-containing/functionalized polymers	Possible thermal-, redox-, and photo-initiation Cell encapsulation/injectability Tunable properties	Risk of cytotoxicity	37, 46, 47
Small crosslinkers (covalent)	Polymers with suitable functionalities (e.g. NH_2_, COOH, CHO) to react with crosslinking agents	Easiness and versatility Tunable properties	Possible cytotoxicity of the crosslinking agent	40, 43, 48
Direct chain–chain crosslinking (covalent)	Polymers functionalized with reactive functional groups	No toxic crosslinking agents Tunable properties	Risk of cytotoxicity Laborious polymer modification step	6, 43
Enzymatic crosslinking (covalent)	Protein-based hydrogels (collagen, gelatin, fibrin, using transglutaminase)	Crosslinking occurring under physiological conditions High cytocompatibility Cell encapsulation/injectability Easily tunable properties	Poor mechanical properties	6, 49–51

ESHU: poly(ethylene glycol)–poly(serinol hexamethylene urethane); PNIPAAm: poly(*N*-isopropylacrylamide); LCST: lower critical solution temperature.

#### Physical crosslinking

In physically crosslinked hydrogels, the junctions among polymeric chains are mediated by transient non-covalent interactions such as ionic interactions, hydrogen bonds, and hydrophobic effects or simply by chain entanglement.^52^ These processes allow to avoid the addition of cytotoxic initiators and chemical crosslinkers and to employ mild conditions of preparation (e.g. pH and temperature) thus improving the cytocompatibility of the hydrogels and possibly permitting the incorporation of cells prior to gelation (e.g. type I collagen gels^53^). Physical crosslinking techniques employed for the preparation of hydrogels rely on (1) ionic crosslinking, where polyelectrolytes form hydrogels in the presence of multivalent ions of the opposite charge that create bridges between pairs of charged functionalities present along the backbone of the polymeric chains,^54^ such as in the gelation process of alginate and pectin by calcium ions;^41^ (2) hydrogen bonding, such as in gelatin-based hydrogels;^42^ and (3) hydrophobic association, occurring when the hydrophobic portions of amphiphilic polymers in aqueous milieu aggregate as the temperature is increased above their transition temperature,^43^ as recently described for the injectable thermosensitive copolymer poly(ethylene glycol)–poly(serinol hexamethylene urethane) (ESHU) that forms hydrogels at body temperature and has been successfully used for bone marrow MSC (BMSC) transplantation.^45^

The main drawbacks of physical hydrogels are generally the low mechanical properties, deriving from the weakness of the secondary forces involved in crosslinking, that limit their application to non-load-bearing sites. In addition, the stability in physiological environments could be an issue given that premature disassembly of the hydrogels can prevent effective cell engraftment.

#### Covalent crosslinking

Covalently crosslinked hydrogels overcome the limitations of physical hydrogels related to stability, dwell time after implantation, and, partially, mechanical properties. Many different chemistries have been employed for covalently crosslinked hydrogels among which are free radical polymerization, click chemistry, Michael-type addition, photocrosslinking, and enzymatic crosslinking.^6,54^ Generally, these systems are composed of polymeric chains bearing reaction sites for 3D network expansion under specific physical and chemical conditions. However, these approaches are suitable for tissue engineering/regeneration only if the employed possibly toxic reagents (precursors, initiators, crosslinkers) can be completely removed before cell addition or implantation. In addition, most of the covalently crosslinked hydrogels do not allow direct incorporation of cells inside the hydrogel, making necessary to seed the cells on the surface and, provided that there is a suitable open macroporosity, let them migrate inside the scaffold. Moreover, chemical functionalization and crosslinking of the starting polymer chains can thoroughly affect their chemistry and then their biological properties, especially for naturally derived materials.

An interesting approach to covalently crosslink polymers in hydrogel systems is to use enzymes. In this approach, the crosslinking reaction proceeds under physiological conditions making the systems highly cytocompatible, injectable for in situ gelation and suitable for direct cell encapsulation. In addition, the properties of the resulting hydrogels can be modulated by controlling the concentration and the activity of the enzymes. One of the most employed enzymes is transglutaminase. Transglutaminases are enzymes that catalyze the formation of isopeptide (amide) bonds between proteins in processes such as blood clot formation. These classes of enzymes have been used to crosslink hydrogels based on different proteins, mainly collagen^49^ and gelatin,^55^ that were demonstrated to be non-cytotoxic and suitable for cell encapsulation.^50,56^

For the in-depth description of crosslinking techniques in hydrogels, the reader is referred to specific comprehensive reviews.^6,52^

#### Controlling hydrogel formation by 3D printing technologies

For many years, hydrogels have been formed as bulks or particles, without any control of the organization of the scaffold. Likewise, inclusion of cells or bioactive factors was achieved by simple blending procedures or surface seeding. The recent development of several 3D printing technologies has opened the way to new possibilities of better controlling the pattern of gels, in particular structure and porosity, from the macroscopic to the microscopic scale, enabling the design of complex, heterogeneous products comprising materials, cells, and growth factors with a controlled organization.^57^ Three types of printing technologies are currently used: inkjet, extrusion, and laser-mediated printing, allowing different resolutions.^57^ Different compounds have been used to produce hydrogels by extrusion and inkjet techniques, such as collagen,^58^ alginate,^59^ silk fibroin,^60^ or synthetic polymers such as polyethylenglycol, acrylates, polyion complex hydrogels,^61^ or polycaprolactones (PCLs).^62^ These technologies also allow the production of interpenetrating networks consisting in the mixture of different polymers resulting in improved overall mechanical properties.^59^ The possibility to perform in situ gel formation upon printing, by physical agents such as ultraviolet (UV) light or temperature, or chemical agents such as pH or radicals,^63^ has been shown to greatly improve the accuracy and stability of the printed pattern.^64^ In addition to controlling gel structure, it is also possible to control cell patterning, using specific natural matrices as bioink for cell printing and separate nozzles to print the gel-forming solution and the cell-containing matrix separately. The combined control of material and cell patterning offers multiple applications for the repair of many tissues, including bone.^65,66^ However, whereas many protocols have shown excellent in vitro properties such as cytocompatibility, well-controlled cell distribution, viability over extended periods of time in culture, and sometimes improved osteoblast differentiation, the real benefit of 3D printing technologies for bone regeneration remains to be demonstrated by further in vivo studies.

### Tailoring hydrogel properties for cell incorporation and bone tissue regeneration

Several material properties must be tuned in parallel to obtain physiologically relevant micro-environments for bone cell incorporation, survival, and differentiation; for bone ECM deposition; and for the recruitment of cells involved in the complex bone regeneration process.

The mechanical properties of hydrogels for BTE are important since scaffolds are supposed to bear loads while promoting the tissue regeneration. In general, hydrogels feature poor mechanical properties, compared to the bone tissue limiting their application to non-load- or low-load-bearing sites.^37^ However, secondary materials such as HA nanoparticles, bioglasses, carbon nanotubes, and nanofibers can be incorporated into hydrogels, obtaining composite materials with appropriate mechanical performances, as recently reviewed by Tozzi et al.^8^ and Butcher et al.^67^

In addition, material stiffness can influence cell behavior in terms of adhesion, proliferation, migration, and differentiation,^39^ with higher matrix rigidity associated with increased osteogenic differentiation of osteoblast progenitor cells and tissue mineralization.^68^ Hydrogel stiffness can be tailored by playing on several preparation processing parameters such as polymer molecular weight, concentration, and type and degree of crosslinking; however, this will also affect other relevant properties of the system, in particular porosity, permeability, and cytocompatibility,^8^ making necessary to finely tune the material design to obtain adequate combinations of mechanical, structural, and biological properties.

When considering hydrogel mechanical properties, polymer degradation should also be taken into account. In fact, ideally, the scaffold should degrade at a rate compatible with new bone formation, so that the mechanical stability of the site of injury is maintained,^69^ and the degradation products should be nontoxic for the cells present in the regenerating and surrounding tissues. Degradation usually occurs by (enzymatic) hydrolysis of ester linkages, and degradation rate can be controlled through the chemistry and length of the polymer backbone and the crosslinkers, through the crosslinking density, and by the introduction of degradation sites susceptible to cleavage by enzymes such as metalloproteinases.^70^

Porosity of hydrogels strongly influences the fate of osteogenic progenitor cells and thus their ability to mediate new bone formation. In general, hydrogels for BTE should have a high and open interconnected porosity to maximize surface-to-volume ratio and thus cell–biomaterial interactions, facilitate cell seeding and colonization, allow the appropriate supply of oxygen and nutrients from the surrounding tissues, and permit neo-vascularization. Porosities higher than 90% are often chosen for BTE scaffolds,^71^ and pore size bigger than 200 µm is generally considered appropriate to stimulate osteogenesis, osteoinduction, osteoconduction, and osteogenic progenitor cell differentiation.^40,72–75^

Porosity can be tailored by playing with the degree of crosslinking (higher crosslinking corresponds to reduced porosity) and porogen materials can be introduced during hydrogel preparation to finely control the final structural properties of the scaffold.^76^ Recently, Wang et al. proposed uncrosslinked gelatin microspheres as porogen agent. Gelatin microsphere can be incorporated in the hydrogel at room temperature but they dissolve in non-cytotoxic products at 37°C, allowing to control porosity and pore size in cell-laden hydrogels without affecting cell viability.^76^

However, it must be noticed that scaffold mechanical strength decreases with porosity and pore size, and therefore these parameters should always be balanced to guarantee the preservation of the mechanical stability of the hydrogel.

The possibility to directly and uniformly encapsulate cells during preparation is a desired property of hydrogels for BTE, since it bypasses the time-consuming cell-seeding/colonization steps necessary for the cellularization of prefabricated scaffolds often associated with limited and unequal cell infiltration. In addition, these systems are often also injectable, allowing minimally invasive administration routes and to easily fit the defect, thus providing a superior configuration for osteoconduction and vascularization from the surrounding tissues.^6^ For direct cell encapsulation, the gelation process must occur in cell-compatible conditions (pH, temperature, osmolarity). When covalent crosslinking is employed, often the chemical reactions have cross-reactivity with cell components, and it is therefore necessary to use nontoxic crosslinkers (e.g. genipin^77^) and initiators (e.g. lithium acylphosphinate salt for photopolymerization) and to investigate the compatibility of the crosslinking strategies with cells, as recently reviewed by Caliari and Burdick.^78^ These issues limit the number of suitable crosslinking strategies and compel to develop specific optimized procedures to preserve the viability of encapsulated cells. Thermosensitive hydrogels are an interesting class of materials that undergo gelation above a transition temperature called lower critical solution temperature (LCST), due to hydrophobic association. Thermosensitive hydrogel-forming polymers are amphiphilic copolymers whose LCST can be tuned by changing the molecular weight of the hydrophobic and hydrophilic portions. When the LCST is at values around physiological temperature, these systems are suitable for cell encapsulation and can be injected into the body for in situ formation.^79^ Poly(*N*-isopropylacrylamide) (PNIPAAm) and Pluronic^®^ are typical examples of thermosensitive polymers that have been used for bone cells’ encapsulations.^80,81^

Biomimetic approaches aim to introduce bioactive molecules in the hydrogel structure to promote osteoconductivity, osteoinductivity, and osteogenicity. First of all, cells used in BTE, in particular MSCs, are strongly adhesion dependent; they need to adhere to the substrate to survive, proliferate, and differentiate; and, when not properly attached, they may undergo anoikis, a form of apoptosis occurring in anchorage-dependent cells when they detach from ECM.^82^ Natural polymers such as type I collagen inherently possess bioactive motifs that can guide cell adhesion, proliferation, and differentiation and tissue regeneration.^83^ When inherent bioactivity is missing, biological cues can be incorporated by covalent grafting or inclusion during fabrication. However, biological moieties’ grafting should be limited to avoid affecting the structural and mechanical properties of the resulting hydrogel. The natural cell binding ligand arginine–glycine–aspartate (RGD), found in collagen, fibronectin, and other ECM proteins, is the most widely employed signal to improve cell adhesion. Interestingly, it has been recently reported that by controlling the distribution of RGD on hydrogels by nanopatterning, it is possible to maximize its beneficial effects on adhesion, survival, and differentiation of MSCs.^84,85^ This strategy is promising for prefabricated hydrogels and can be exploited for the investigation of the influence of RGD density and spatial distribution on in vitro MSC differentiation, but it is hard to translate to hydrogels for BTE applications, especially to hydrogels for direct cell encapsulation, where only the grafting degree can be controlled during synthesis.

As recently reviewed by Nyberg et al.,^86^ growth factors such as BMP-2, TGF-β, FGF, and IGF can be incorporated to control MSC differentiation and the recruitment of progenitor cells from the surrounding systems, aiming to mimic the signaling events occurring during bone healing, and also alternative small osteogenic molecules such as melatonin, resveratrol, and purmorphamine have recently demonstrated promising activity in BTE strategies.^87^ However, fine tuning the properties of hydrogel-based scaffolds for the combined transplantation of cells and controlled delivery of osteogenic molecules is challenging due to the different characteristics required for the two approaches (additional drug delivery particles could be necessary), and it is not clear yet whether the synergistic effects are significant.^88,89^

It is worth to note that, unfortunately, to date the majority of the studies on bone progenitor cell differentiation in hydrogels have been performed in in vitro settings, whose predictivity toward in vivo outcomes is still debated.^38^ In addition, controlling separately structural, mechanical, chemical, and biological properties of hydrogels is challenging since, as described above, usually a change in processing parameters strongly influences these properties all at once, for example, increasing crosslinking density to improve the strength of the scaffold yields reduced pore size and longer degradation times and can affect the cytocompatibility necessary for cell encapsulation. Consequently, also the investigation of the effects of single parameters on cell behavior is not trivial, and the hydrogel system properties as a whole must be taken into account in the design of hydrogels for BTE.

### Hydrogel-forming materials

On the basis of the origin of their components, the materials for the preparation of hydrogel scaffolds can be classified as natural and synthetic. However, to combine the advantages of these two systems, to overcome their limitation, and to obtain optimized properties for tissue engineering, several combinatorial approaches have also been thoroughly implemented.^37^ The advantages and limitations of the main materials employed in hydrogel-based BTE are summarized in Table 2, together with relevant examples of their in vitro and/or in vivo performances.

**Table 2.** Main hydrogel-forming materials used in bone tissue engineering.

Hydrogel material	Origin	Relevant properties	Limitations	Ref.	Relevant studies in BTE
Collagen	Mammals (natural)	Main protein of bone ECM Injectability Cytocompatibility Bioactivity FDA approved	Limited mechanical properties	53, 54, 71, 83	In vitro: collagen + BMSCs/umbilical cord MSCs: osteogenic differentiation and mineralization^90^
Alginate	Marine algae (natural)	Cytocompatibility Easy chemical modification Tunable properties	Must be preformed to be injectable Low cell adhesionLimited mechanical properties	41, 54, 91	In vitro, in vivo: RGD-grafted alginate + endothelial cells: mineralized ECM deposition^92^ In vivo: alginate + demineralized bone ECM: ectopic bone mineralized ECM deposition + vascularization^93^
Chitosan	Exoskeletons of crustaceans (natural)	Antibacterial activity Biocompatibility Easy chemical modification Tunable properties	Limited mechanical properties	94, 95	In vivo: chitosan + BMSCs and hydroxyapatite: calvarial bone repair^96^
Pullulan	Fermentation of starch by a fungus (natural)	Cytocompatibility FDA approved	Low cell adhesion	97, 98	In vivo: cholesterol-bearing pullulan + BMP-2: new bone formation in calvarial defects^99^
PEG/PEO	Synthetic polymers	Easy chemical modification Tunable properties Biocompatibility FDA approved	Low cell and protein adhesionLow degradation rate	70, 100–102	In vitro: inkjet bioprinted photopolymerized PEG + RGD + BMSC: osteoblast differentiation and scaffold mineralization^46,65^ In vivo: thermosensitive PEG-*b*-polycaprolactone copolymers + hDPSCs: osteoblast differentiation and mineralized ECM deposition^103^
PPE	Synthetic polymers	Auto-calcification promotion by degradation products Tunable properties	Fast degradation rate	70, 104–106	In vitro: photocrosslinked PPE + BMSCs: cell survival and deposition of mineralized ECM^107^
Peptides	Amino acid sequences (synthetic)	Cytocompatibility Non-immunogenicity Bioactivity Tunable properties Physical crosslinking induced by ionic force, pH, or temperature	Low mechanical properties	108–110	In vitro: RADA16^®^ peptide hydrogel + BMSCs: osteoblast differentiation and mineralized bone ECM deposition^111^ In vivo: PuraMatrix™ hydrogel + PEEK cage: bone regeneration in bone defects^112,113^

BTE: bone tissue engineering; ECM: extracellular matrix; FDA: Food and Drug Administration; BMSCs: bone mesenchymal stromal cells; MSCs: mesenchymal stromal cells; RGD: natural cell binding ligand arginine-glycine-aspartate; BMP: bone morphogenetic protein; PEG: polyethylene glycol; hDPSCs: human dental pulp stem cells; PPE: polyphosphoester; PEEK: polyetheretherketone.

#### Natural hydrogels

The main naturally occurring polymers synthesized by living organisms are polysaccharides (e.g. pullulan, alginate, chitosan) or proteins (collagen, silk, fibrin, heparin, etc.).^39^ In general, natural polymers are biocompatible and they allow cell attachment and proliferation due to their physical and biological properties without causing cytotoxic reactions compared to synthetic hydrogels.^39^ The main limitations of these polymer-based matrices are their lack of mechanical strength to support the forces occurring in the bone environment and, for some of these polymers, a lack of cytocompatibility and osteoconductivity, fast degradation rates, high batch-to-batch variability, and some immunological concerns.

##### Collagen

Type I collagen hydrogels are prepared from collagen extracted from mammals by simple neutralization of the acidic collagen solution.^53^ Their thermosensitive nature allows the cells to be incorporated when they are liquid, at low temperature, and then to be injected and form a gel in situ, at 37°C. Collagen is an important constituent of the bone ECM, and therefore it offers a number of favorable binding sites for bone cells and it is reported to promote mineralized matrix deposition.^72,114,115^ These physical and biological properties make collagen a very good candidate for BTE as evidenced by the high number of papers present in the literature, although the mechanical properties of collagen-based hydrogels are fairly limited.^37,83,116^ In addition, recently, collagen gels have been described as optimal scaffolds for the coculture of MSC and endothelial cells, promoting, in vitro, the increased expression of both osteogenic and angiogenic markers, with respect to other systems such as alginate gels.^117^

##### Alginate

Among natural polysaccharides, alginate is a linear anionic copolymer composed of (1–4)-linked β-d-mannuronate and α-l-guluronate residues that is extracted from marine algae.^118^ Usually, alginate hydrogels are formed by crosslinking of the hydrophilic polymer by n (Ca^2+^).^41^ Alginate is generally employed to encapsulate cells and/or molecules such as small chemicals, proteins, and drugs.^94^ However, to promote cell adhesion and proliferation, an improvement of the hydrogel composition is necessary such as the chemical grafting of RGD-containing peptides on the polymer backbone.^41^ As an example, Grellier et al.^92^ showed that BMSCs can synthesize both in vitro and in vivo a mineralized ECM in RGD-grafted alginate microspheres.

##### Chitosan

Chitosan is a linear polysaccharide consisting of randomly distributed *N*-acetyl-d-glucosamine and d-glucosamine units linked by β (1 → 4) glycosidic bonds. Chitosan is obtained by the deacetylation of chitin, one of the main components of the exoskeletons of crustaceans and the cell walls of fungi. Due to the presence of deacetylated units, chitosan is protonated at slightly acidic pH, and these characteristics confer it many of its peculiar properties such as the ability to form hydrogels by ionotropic gelation and antibacterial activity.^118^ In addition, depending on the chemical properties of the used chitosan and on its eventual derivatization, chitosan-based hydrogels can be prepared by chemical crosslinking, allowing to obtain a wide range of mechanical, thermal, and biological properties^119^ and in situ gelation can be achieved by photocrosslinking with UV light or temperature-induced crosslinking.^95^ To promote cell adhesion and proliferation, the degree of deacetylation of chitosan should be high and this is also reported to improve the mechanical properties of the obtained hydrogels.^120^ In a recent approach, Ding et al.^121^ deacetylated preformed chitin hydrogels yielding physical crosslinked hydrogels with superior mechanical properties, increasing with the degree of acetylation. The reported mechanical and structural properties are promising, but the ability of these systems to promote bone tissue regeneration still needs to be investigated.

##### Pullulan

Pullulan is another neutral and non-immunogenic polysaccharide produced from the fermentation of starch by the fungus *Aureobasidium pullulans*. It is composed of maltotriose units (blocks of three glucose residues connected by α-1,4 glycosidic bonds) connected to each other by α-1,6-glycosidic bonds. To improve the mechanical stability of pullulan, it can be crosslinked by trisodium trimetaphosphate which is reported to be nontoxic.^122^ Unfortunately, notwithstanding the hydrophilic nature of pullulan-based hydrogels, they do not support adhesion and spreading of cells. To overcome this problem and enhance biostability, pullulan has been combined with other materials such as gelatin or HA nanocrystals^97^ or coated with bioactive proteins such as silk fibroin.^98^

#### Synthetic hydrogels

Synthetic hydrogels offer many advantages over natural polymers including unlimited supply, relative lack of immunological concerns, and much higher reproducibility in terms of physical and chemical properties, which is important for the reproductive manufacture of tissue engineering/regeneration products. In addition, they offer the potential for improved control and tuning of the properties, repeatability, and safety.^39^

Currently, synthetic polymers have emerged as an important alternative for the production of hydrogel-based scaffolds for BTE.^108^ Because of their synthetic nature, the chemical properties of these polymers can be easily tailored ad hoc to adapt and modulate their physicochemical properties to obtain hydrogels that better mimic the morphology and mechanical properties of native extracellular matrices or to modify their kinetics of biodegradation.^123^ In addition, they can be functionalized with bioactive compounds to improve their biomimetic and osteogenic behavior.^124^ Among the many synthetic polymers available to create hydrogels, only few of them have the properties necessary to be selected as physiologically relevant micro-environments for BTE, such as polyesters (e.g. polyglycolic acid, polylactic acid (PCL)), polyacrylates, polyethylene glycol (PEG), polyphosphoesters, and synthetic peptides.

##### PEG-based hydrogels

PEG, also known as polyethylene oxide (PEO), is a linear polyether manufactured from ethylene glycol monomers. The name PEG is usually used to indicate polymers with a molecular weight lower than 20 kDa while PEO refers to chains with higher molecular weight. For the preparation of hydrogels, PEG is usually crosslinked by gamma-irradiation or chemical crosslinking by reaction of hydroxyl groups on the ends of PEG^125^ or upon previous functionalization with other functional groups.^6,108^ PEG-based synthetic hydrogels are among the most studied and employed systems for protein and cell delivery in regenerative medicine because of their good and tailorable mechanical properties, high biocompatibility, and low immunogenic profile; furthermore, PEG is commercially available and Food and Drug Administration (FDA) approved for several applications and it can be easily functionalized incorporating the desired functionalities.^100^ Photocrosslinked diacrylate PEG (PEGDA) hydrogels have shown interesting properties. In the study by Nuttelman et al., MSCs were encapsulated within the hydrogel before photocrosslinking and could survive and differentiate into osteoblasts. In particular, cells expressed markers of osteoblastic differentiation such as osteonectin, osteopontin, and ALP, and a mineralization of hydrogels was observed using von Kossa staining.^126^

PEG, notwithstanding its high hydrophilicity, is recognized as a biologically inert polymer, consequently both protein and cell adhesion can be fairly limited in PEG-based hydrogels. A frequently employed strategy to improve cell attachment, proliferation, and, potentially, differentiation on these substrates consists of the functionalization of PEG hydrogels with RGD motif-containing peptides.^8^ Several studies have shown that the incorporation of RGD adhesive peptides increased osteoblast and MSCs’ attachment, survival, proliferation, and differentiation;^65^ furthermore, the mineralization of hydrogels^46,65^ and the production of bone tissue marker proteins were ameliorated.^127^

The tri-block copolymer commercially known as Pluronic F127, made of amphiphilic copolymers PEO and polypropylene oxide (PPO), (PEO)_99_–(PPO)_69_–(PEO)_99_, can form synthetic hydrogels. Pluronic is characterized by a thermoreversible gelation: it is liquid at 4°C and forms gel within 5 min at 37°C.^128^ In addition, it has favorable properties such as biocompatibility, non-cytotoxicity, and biodegradability.^81^ The study conducted by Diniz et al. showed that this hydrogel allows adhesion, survival, and proliferation of human BMSCs and dental pulp stem cells seeded within the hydrogel. In addition, the authors have shown that when cellularized hydrogels are cultivated in an osteogenic medium, cells express osteogenic differentiation markers and deposit mineralized bone ECM, making Pluronic a good candidate for BTE.^81^ Despite this, the biostability properties and the too rapid degradation in aqueous environment of this hydrogels still limit their use as cellularized scaffolds in vivo.^81,129^

A valuable alternative are methoxy-PEG-*b*-PCL (MPEG-PCL) block copolymers that have thermoresponsive properties similar to Pluronic, low degradation rates, and suitable mechanical properties and in the last few years have been successfully tested for in vivo osteogenic potential in combination with MSCs from different origins.^103,130^

##### Polyphosphoester-derived hydrogels

Polyphosphoesters are a class of phosphorus-containing polymers featuring repeating phosphoester bonds in their backbone. Polyphosphoester-based hydrogels, by choosing the appropriate starting monomers, (macro)crosslinkers and initiators, can be synthesized by photoinitiated free radical co-polymerization in mild physiological-like conditions and combined with other polymers such as PEG.^104^ In addition to their biocompatibility, anyway still dependent on the choice of the building blocks, an interesting property of polyphosphoester hydrogels is related to the degradation products; in fact, the hydrolysis of phosphate linkages produces phosphate, alcohols, and diols, with low cytotoxicity.^70,107^ In addition, phosphate reacts with the calcium ions present in the surrounding environment producing calcium phosphate, thus promoting auto-calcification which may further stimulate cells toward bone ECM deposition.^105^

It has been shown that MSCs seeded in polyphosphoester-based hydrogels and incubated in an osteogenic medium survived and led to mineralization of the hydrogel after 3 weeks of culture.^107^ The major limit of these hydrogels is a too rapid weight loss over time in culture due to the presence of numerous cleavage sites available for enzymatic biodegradation, leading to a degradation profile that, if not well tuned by optimizing the chemistry of the polymer, may be too fast for the occurrence of an adequate bone regeneration.^105^

##### Peptide-derived hydrogels

Synthetic peptide–derived hydrogels are formed by relatively short (around 15–20 residues) amino acid sequences (i.e. self-complementary peptides and peptide amphiphiles) capable of self-assembling into hydrogel networks by physical crosslinking induced by ionic force, pH, or temperature changes.^108^ These hydrogels show biocompatible, biodegradable, and generally non-immunogenic properties; moreover, their nanofibrous network organization mimics the natural ECM fibrillar structure.^108^ An in vitro study of BMSCs seeded within a commercial peptide hydrogel (RADA16^®^) grown in an osteogenic medium has shown encouraging results for BTE. Indeed, progenitor cells in the hydrogel differentiated into mature osteoblasts and a high and increasing activity of ALP and osteocalcin contents were observed after 2, 3, and 4 weeks of maturation.^111^ For in vivo use in BTE, the mechanical properties of these hydrogels appear to be low, but some possibilities have proposed to overcome this limitation,^109,110^ especially based on chemical crosslinking; however, it must be ensured that these modifications do not affect the good cytocompatibility and the osteoinduction/osteogenic properties of these hydrogels.^110^

#### Multicomponent hydrogels

As we have seen, the existing hydrogels for BTE need to compromise between good biological properties (cell attachment, proliferation, and differentiation) and good mechanical properties (mechanical resistance to environmental stresses and controlled degradation). Combining different materials is a strategy that may permit to obtain simultaneously biological activity and mechanical support. As mentioned previously, natural hydrogels synthesized from natural polymers such as proteins usually have structures and biological properties which actively regulate cellular responses, offer favorable interactions with the surrounding ECM, and promote osteogenesis. Oppositely, synthetic hydrogels are often associated with higher mechanical properties and biostability. Consequently, the combination of the characteristics of the synthetic and natural polymers to design hybrid hydrogels is envisaged as a promising approach for the creation of bioactive scaffolds for BTE.^108^ One of the most employed approaches involves the combination of natural polymers with PEG. Coupling a biological molecule to PEG usually contributes to improve the biological activity of the synthetic polymer and confers to otherwise biologically inert PEG hydrogels, cell–protein adhesion properties.^125^ Several studies combined PEG with naturally derived ECM components such as collagen^131^ or hyaluronic acid^132^ and showed enhanced biological properties with high (encapsulated) stem cells viability in vitro and in vivo associated with enhanced mechanical properties.

Pullulan has been recently blended with dextran and sodium carbonate as porogen reagent to form hydrogels with interconnected pores of 200 µm that were seeded with MSCs. These systems demonstrated superior osteogenesis in vivo in a rat model of large bone defect with the hydrogel that was rapidly resorbed and substituted by a dense mineralized bone tissue forming from the edges of the defect. MSCs promoted both bone formation and vascularization, but it was not clear whether they exerted a paracrine effect, a direct bone tissue deposition activity through their differentiation into mature osteoblasts, or a combination of the two processes, since their number was extremely decreased 30 days after implantation.^133^

Another example is PNIPAAm, a temperature-responsive polymer which has the abilities to form hydrogel when heated at 32°C in water. This synthetic hydrogel has been shown to be a good candidate for the encapsulation of bone cells,^134^ but its use is limited because of its poor biocompatibility and non-biodegradability.^135^ To overcome these limitations, many researches modified this hydrogel with natural compounds including collagen,^136^ chitosan,^137^ hyaluronic acid,^138^ or RGD peptides.^139^ Liao et al. have shown that hyaluronic acid–chitosan–PNIPAM hydrogels can promote MSCs’ proliferation and osteogenic differentiation and secretion of mineralized ECM after culture in an osteogenic environment. Also in vivo grafting of the injectable hydrogel–MSCs’ complex demonstrated ectopic bone formation and total biodegradation of the material without toxic reaction to animals.^140^

#### Composite hydrogel

Composite hydrogels aim to combine natural or synthetic hydrogels with bioactive phases, degradable polymeric structures, and/or bioceramics to enhance the mechanical and biological properties of each compound to produce a relevant environment for BTE.

To increase osteoinductive, osteoconductive, and mechanical properties of hydrogels, one strategy consists of loading them with usually micro/nano-sized mineral phase-like ceramics to promote tissue formation while providing higher initial mechanical properties to bear the solicitations occurring in the bone environment.^115^ Thus, new composite matrices combining polymers and calcium phosphates have been developed to mimic as closely as possible the bone matrix, a mixture of organic and inorganic components. Elements based on calcium phosphates provide mechanical properties and osteoconductivity, and the polymer component, collagen, chitosan, or alginate, improves the biocompatibility and biodegradability of the biomaterial.^8^ Three of the most commonly used mineral supplemented matrices in BTE are calcium phosphate ceramics, tricalcium phosphates, and HA. These mineral compounds show adequate biocompatibility and suitable osteoconduction and osseointegration properties.^141^ In one of these biomimetic approaches, a composite hydrogel of type I collagen and HA could enhance osteoblast differentiation^142^ and accelerate osteogenesis.^143^ Alginate hydrogels blended with HA were reported to support the adhesion and proliferation of osteosarcoma MG-63 human cell line. The system showed at the same time adequate structural and physical–chemical properties for being used as scaffolds in BTE strategies but it is not injectable.^144^

It must be noted that in general, the mechanisms of interaction between hydrogel networks and the supplemented inorganic particles still need to be elucidated in depth. Data about in vivo applications of these systems are still limited, making necessary further comprehensive studies on the long-term performances, cytotoxicity, biocompatibility, biodegradability, and osteogenic activity of such composite hydrogels under in vivo conditions to confirm the promising properties of this class of materials for BTE.^145^

Cellularized hydrogels can also be used as fillers of degradable porous polymeric structures serving as bone grafts that temporarily bear loads. Heo et al.,^146^ for example, have recently combined 3D-printed polymeric porous microstructures with photo-curable gelatin hydrogels laden with adipose-derived stem cells (ADSCs) that demonstrated osteogenic capability in vitro.

## Choosing and preparing cells for BTE

### The different cell types used for BTE

Regenerating bone in areas where no/few suitable progenitor cells are available to differentiate and synthesize and deposit an osteoid matrix requires the input of exogenous cells which, associated with an appropriate scaffold and other factors, will differentiate into functional osteoblasts, the primary actors of bone formation. The choice of the source of osteoblast progenitors and the procedure used to isolate, amplify, and prepare them before seeding the 3D scaffold and grafting the construct in the host site have significant consequences on the efficiency of the BTE product. This choice must consider several parameters and will usually result from a compromise between advantages and drawbacks.

#### Adult mesenchymal stromal cells

Mesenchymal stromal cells (MSCs) are the most widely used stem cells for BTE applications. This statement is supported by the large predominance of publications where the keywords “bone tissue engineering” are associated with the word “MSCs” (5539 articles and 700 reviews found in PubMed^147^) over association with other cell types (492 articles and 181 reviews for embryonic stem cells (ESCs) and 191 articles and 66 reviews for induced pluripotent stem cells (iPSCs)). Moreover, there are currently 24 clinical trials ongoing for the treatment of bone fractures that use MSCs whereas none is so far reported using ESCs or iPSCs.^148^

MSCs used for BTE have been obtained from several tissues and organs, including bone marrow,^1^ adipose tissue,^149,150^ amniotic fluid,^151^ dental pulp, or Wharton’s jelly.^152^ A specific and selective cell surface marker for the MSC has yet to be determined, but these cells are typically identified by their expression of CD90, CD105, CD73, and CD146 and absence of CD45, CD34, CD14, CD11b, CD79a, CD19, and HLA-DR. Without clear markers for cell sorting, the International Society for Cellular Therapy has proposed a set of basic requirements for a cell to be classified as an MSC. MSCs are defined as a plastic culture adhesive cell with the ability to generate a colony-forming unit and differentiate into bone, cartilage, and adipose tissues.^153^

MSC from different origins show important similarities in their transcriptome profile, but significant differences in the expression of a subset of genes have been observed.^154,155^ These differences in gene expression have not been correlated with functional differences, but clearly demonstrate that MSC identity depends on their origin and suggest that the origin may explain the phenotypic differences observed upon in vitro and in vivo manipulation of these cells. Several studies have reported differences in proliferation and differentiation capacity of MSC according to their tissue origin, when they are grown in identical conditions in vitro. Concerning proliferation capacity, no consensus emerges; some studies reporting, for instance, a higher proliferation rate for BMSCs over ADSCs,^156^ while others showing the opposite behavior.^157^ The differentiation potential under identical culture conditions yields a clearer picture, with a significantly higher osteoblastic differentiation potential for BMSC over ADSC, for instance, reported by many studies under different micro-environments.^156,157^ The more recently characterized dental pulp–derived stem cells exhibit superior osteogenic properties as compared to the two previously mentioned MSC subsets, and this increased capacity to differentiate into osteoblasts has been correlated with improved bone formation in vivo.^158^ The capacity of MSCs to elicit endothelial cell differentiation is limited regardless of MSC origin; however, BMSCs seem to have a higher potential.^159^

At this point, the choice between these different sources of MSCs may be determined not only according to the differentiation capacities but also to the easiness of cell harvesting and amount of cells that can be collected. In this respect, adipose tissue should be ranked first, for its easy access and high proportion of MSCs within the stromal vascular fraction, obtained by digesting the fat and concentrating the remaining cells. Isolation of cells from dental pulp, although the proportion of MSCs is very high, requires wisdom tooth extraction, which is not really applicable to most patients. Other sources of stem cells are also compromised by the difficulty to obtain them or by their low quantity and are therefore likely to remain models used in fundamental research for the studies of stem-cell differentiation and repair capacity, without real therapeutic applications. For instance, MSCs from cortical bone show a very high osteoblast differentiation capacity and in vivo osteogenic potential,^160^ but the difficulty to obtain them rules them out any therapeutic perspective. Likewise, periosteal stem cells are perhaps the most relevant to bone regeneration since they are the primary source of cells that heal the fracture,^161^ but these cells are unlikely to play a significant role in therapeutic strategies since periosteal stripping could negatively impact normal bone homeostasis and cause donor site morbidity. These cells are also difficult to access and available in low quantity.

Two major drawbacks of MSCs are heterogeneity^162^ and donor-dependent variability.^163^ A promising route to improve the efficiency of MSCs is to select and isolate specific sub-populations, using cell sorting based on specific markers.^164,165^ For instance, a recent study by König et al.^166^ showed the superior bone forming capacity of CD146+ pericytes from fat tissue. However, this selection procedure is likely to reduce the amount of cells produced from the biopsies and available for clinical applications.

In addition to their osteoblast differentiation capacity, MSCs play an important role in regulating inflammation and have a trophic function in stimulating tissue regeneration.^167^ The immunomodulatory role of MSCs plays a critical role both in normal healing and in therapeutic approaches. Immunomodulation by the MSCs is accomplished by secretion of immunosuppressive and anti-inflammatory cytokines, such as IL-10, nitric oxide, and prostaglandins. MSCs can also regulate T cells in an antigen-independent manner through the suppression of the primary and secondary T-cell responses by inhibiting cell proliferation. MSCs also promote a local healing response by stimulating proliferation and differentiation of resident stem cell populations, reducing fibrosis, and inhibiting adverse apoptosis. MSCs secrete several cytokines such as TGF-β, stem-cell factor (SCF), IGF, epidermal growth factor (EGF), and granulocyte and macrophage colony-stimulating factors (G/M-CSFs). Taken together, the immunosuppressive and trophic capabilities of MSCs are powerful and may play an important part in the tissue regeneration process. Of particular interest is how MSCs appear to have a lasting therapeutic effect despite a transient persistence after engraftment. Indeed, a major limitation of MSCs is their short lifespan after implantation. In many studies where MSC number is monitored, more than 90% of the grafted cells die within 14 days.^168,169^ Anoikis has been shown to be a major cause of cell death when cells do not adhere to the matrix to which they have been associated.^170^ Ischemia is another major cause for the death of grafted cells,^171^ and the association of oxygen carriers with BTE constructs has been shown to improve survival and the bone regeneration capacity of the cells.^172,173^ The availability of glucose is another key parameter, and combinations of low glucose and low oxygen result in severe cell loss.^174,175^

##### Influence of the procedures used for isolation, maintenance, and conditioning of MSCs

The production of cells from human tissues involves several steps. The isolation procedure involves mechanical action, sometimes enzymatic digestion, and always an abrupt change on the physical and biochemical environment. Indeed, cells are suddenly transferred from a soft and relatively hypoxic micro-environment in their tissue of origin into a solution under 21% oxygen during the time of dissociation, and then transferred again onto a very stiff plastic culture dish in a special culture medium. Modification of matrix stiffness^176–178^ and oxygen concentration^179^ has been extensively shown to critically affect cell phenotype. The transient exposure of cells to a very stiff substrate (such as plastic) has been shown to favor osteoblast differentiation that was maintained even after cells were transferred to a softer substrate, such as 3D matrices for implantation.^176^ Following this stressful treatment, they will be usually grown for several days and will often have to undergo a few passages, involving cycles of trypsinization and re-plating. Although the consequences of each of these steps have not been examined thoroughly, some studies report significant consequences of the procedures used to isolate and grow cells prior to their embedding in the host 3D matrix which will be used for engraftment. The protocols used for harvesting MSCs from bone have been shown to affect their capacity of differentiation toward osteoblasts.^180^ Plating and passages have been shown to alter the pattern of expression of several surface markers^181^ and more generally affect their transcriptome.^182^ Such modifications seem to affect the capacity of MSCs, once seeded into a 3D matrix and implanted in host tissues, to differentiate into osteoblasts and, most importantly, to efficiently produce a bone matrix.^183^ Interestingly, treatment of cells with melatonin during this expansion period has been proposed to preserve their differentiation capacity.^184^ Given the loss of performances of MSCs upon long-term culture, it is advisable to reduce the number of passages (usually MSCs undergo less than five passages before engraftment), although a compromise must be found between a large number of cells, requiring prolonged amplification and maintenance of therapeutic efficiency of cells. A recently proposed alternative to expansion in two-dimensional (2D) culture of MSCs is their growth as spheroids. MSCs spontaneously associate to form these structures when they are grown on a low-adhesive substrate. These structures evolve within hours from a loose aggregate toward a compact sphere. Interactions between cells are much more abundant in these structures than in 2D cultures, and cells are exposed in different environments according to their position within the spheroid. Several studies have shown that the phenotype of MSCs in spheroids differs in many aspects from the one of cells grown in 2D: stemness, differentiation capacities, immunomodulatory, and anti-inflammatory effects are enhanced in 3D aggregates.^185^ In terms of bone regeneration potential, spheroids have been shown to favor osteoblast differentiation, especially in the absence of osteoinductive factors in the culture medium. They also exhibit increased secretion of v-EGF, potentially favoring the vascularization of newly formed bone.^186^

#### Human ESCs and iPSCs

The capacity of human ESCs to produce bone matrix has been tested with different protocols and scaffolds. Two strategies have been explored, the direct differentiation of ESCs toward osteoblasts and the prior differentiation toward MSCs, yielding ESC-derived MSCs, which were subsequently driven toward the osteoblastic lineage.^187^ Direct differentiation of ESCs into osteoblasts was achieved using osteoinductive scaffolds and the classical osteogenic culture medium. Studies have shown a higher proliferation rate with ESCs than with MSCs, favoring the colonization of the scaffold.^169^ However, ESCs show notable tumorigenic properties: they are characterized by high telomerase activity (which leads to potentially infinite proliferation) and are known to form teratomas.^188,189^ Nonetheless, ESCs’ handling is surrounded by several ethical issues due to their embryonic provenance, thus making improbable their use in bone defects’ treatment, at least in the near future.

The use of iPSCs for BTE has emerged after the initial description of the reprogramming of different human somatic adult cells.^190^ To avoid tumorigenic potential of these cells, they have to be pre-differentiated toward the mesoderm lineage before being implanted. This can be achieved either via the formation of intermediary embryoid bodies or directly from isolated cells.^191^ In all cases, different osteogenic media were used, either supplemented with β-glycerol phosphate, ascorbic acid, and dexamethasone or with growth factors such as TGF-β, insulin growth factor-1 (IGF-1), basic FGF-β, or BMP-2, and they showed to enhance the osteogenic capability of iPSCs. The types of scaffolds used to grow, differentiate, and implant iPSCs were not different from those used for MSC, including natural or synthetic polymers or combinations of both, sometimes combined with an osteoconductive HA component.^192^ An important issue, as for MSCs, was to compare the osteogenic capacity of iPSCs according to their tissue of origin. A few studies report improved osteogenic properties of iPSCs derived from bone marrow stromal cells as compared with other sources, both in vitro and in vivo.^193^ Although cells generated from bone marrow exhibited a higher osteogenic potential, all sources were used successfully to produce bone tissue. Several studies have also shown that bone formation could be obtained either by the direct use of iPSCs or via prior formation of embryoid bodies.^194^ These cell sources seem promising, but it remains to be checked that implanted cells do not form teratomas on the long term.

The great advantage of using iPSCs or ESCs compared to MSC is that these pluripotent cells can be grown for a considerable period before differentiation is induced. They maintain their pluripotent property during this amplification step, and only afterward can they be induced toward the osteoblastic differentiation pathway.^195,196^ Besides, it is possible to generate different specialized cell types from a single source of iPSCs, enabling the design of more complex TEPs. For instance, Jeon et al.^197^ have shown that co-implanting osteoblasts and osteoclasts obtained from iPSCs in a HA-coated poly(lactic-co-glycolic acid)/poly(l-lactic acid) scaffold matrix elicited enhanced ectopic bone formation.

### Creating an environment to favor angiogenesis

The efficiency of the tissue engineering products largely depends on their capacity to be rapidly colonized by blood vessels to ensure oxygen and nutrient supply to the embedded cells. Consequently, several strategies have been developed to favor angiogenesis around and within the implanted tissue engineering constructs. Two types of factors determine the efficiency of blood vessel colonization of the scaffold: (1) the macroporosity, which must be sufficient to enable the progression of new blood vessels and, as described above, largely depends on the structure of the hydrogel and (2) the angiogenic potential of the scaffold itself. This potential can be enhanced by the release of angiogenic growth factors by the scaffold (or by their secretion by the embedded cells).

v-EGF is a potent angiogenic factor.^198^ This protein is produced by several cell types including MSCs,^199^ but the secretion of v-EGF by these cells largely depends on the amount of grafted cells, culture conditions,^200–202^ tissue origin,^159^ and other environmental factors, limiting its potential role in neo-angiogenesis. A few studies have addressed the possibility to deliver v-EGF at the site of implantation of biomaterials to stimulate angiogenesis from the host tissue, either alone or in combination with other growth factors such as BMP-2.^203^ The results obtained with these strategies show a limited positive effect of v-EGF on the amount and quality of newly formed bone. This modest input by v-EGF may be due to the fact that v-EGF efficiency is dependent on several parameters such as spatial distribution, association with matrix proteins, and time-dependent availability.^204^ Consequently, strategies aimed at controlling the release of v-EGF have shown improved efficiency,^205^ but they should be further developed. Some of these limitations may also be overcome by the use of transgene-mediated v-EGF production,^203^ but this requires the prior infection or transfection of cells before their association with the scaffold, a procedure that raises additional safety and regulatory issues for therapeutic applications.

An alternative to the use of growth factors to promote angiogenesis is to incorporate endothelial cells or endothelial cell progenitors in the hydrogels, eventually in combination with other cell types such as MSCs or with growth factors. Two types of endothelial cell sources are essentially used: mature endothelial cells generally isolated from umbilical cord vein (human umbilical vein endothelial cell (HUVEC)) or endothelial progenitor cells (EPC) isolated from blood. Because of the possibility to isolate EPCs from the patient to perform autografts, EPCs represent a more attractive source of cells and are therefore preferred in most of the recent studies. EPCs alone, when associated with different scaffolds and implanted in different tissues, have been shown to trigger angiogenesis.^152,206^ However, they do not promote bone formation by themselves. Coculture followed by co-implantation or direct co-implantation of MSCs and EPCs has been shown to result in enhanced osteogenesis, as compared with MSCs alone, but not significant differences in angiogenic properties of EPCs alone. These studies support the notion that MSCs do not enhance the capacity of EPCs to make new blood vessels. Instead, they show that EPCs potentiate the capacity of MSCs to elicit bone formation.^207^ Noteworthy, some studies have shown that using differentiated osteoblasts instead of undifferentiated MSCs in coculture with endothelial progenitors favored blood vessel formation.^208^ Moreover, osteoblasts have been shown to stimulate angiogenesis by the host tissue.^209,210^ It is, however, difficult from the available data to determine whether bone formation is increased because more blood vessels irrigate the scaffold and favor cell viability and function, or whether this synergy results from early, direct cell–cell interactions between MSCs and EPCs. Several in vitro studies have shown direct interactions between these two types of cells, in both 2D^211^ and 3D cultures,^212^ and stimulation of MSC differentiation toward the osteoblastic phenotype. Thus, contact between both cell types during the pre-culture period or within the scaffold upon implantation is probably mandatory to promote increased bone formation. In this context, a new approach consists of the generation of scaffolds integrating osteogenic and angiogenic niches in the hydrogel structure. Photolithography was used by Kazemzadeh-Narbat et al.^213^ to control the photocrosslinkable hydrogel stiffness and the patterned distribution of ECs, MSCs, and preosteoblasts, allowing to obtain, in vitro, mineralized regions surrounded by organized vasculature. The interesting results reported in this proof-of-concept study, performed on a construct with planar geometry, are very promising for the translation of this approach to more complex shapes and for the possible application for treatment of bone defects.

### Pre-conditioning cells before engraftment

Cell fate is determined by the combination of several biophysical and biochemical parameters.^214^ Usually, cells are amplified on tissue plastic dishes in a basal, non-osteogenic medium. However, after embedding into the implantable 3D scaffold, cells can be submitted to very different micro-environments, which significantly affect their in vivo fate.^215^ The parameters of this environment include the following options: incorporation of growth factors, using either the scaffold itself as delivery system or intermediate carriers such as nanoparticles;^216^ the incorporation of HA particles with different physical characteristics; pre-differentiation, or not, before implantation; perfusion of the cellularized scaffolds in bioreactors; and control of oxygen concentration. Combinations of the above-mentioned parameters offer the possibility to create an infinity of different micro-environments.

Whether the cells should be pre-differentiated or not before their implantation is an important issue. Pre-differentiating MSCs toward osteoblastic lineage has been shown to improve their bone formation potential and also their survival after engraftment.^217^ However, some studies have shown that this pre-differentiation step reduces their intrinsic angiogenic properties.^218^ When grown as spheroids, MSCs have an enhanced capacity to differentiate into osteoblasts without osteogenic culture medium; however, the capacity of these structures to elicit bone regeneration is limited.^219^ Although it may improve cell efficiency, pre-differentiation implies prolonged culture time and hence increased risks of contamination and mutations and higher costs, all parameters which are not desirable for therapeutic applications. Therefore, association with osteogenic growth factors, culture in hypoxic conditions, and short-term mechanical stimulation are promising alternatives which are presented herein below.

#### Growth factors

As described above, prominent growth factors involved in bone formation and repair are TGF-β, BMPs, FGFs, EGF, IGFs, and PDGF. The growth factors of the TGF-β super family such as BMPs induced primary signal to upregulate mineral-depositing osteoblasts’ differentiation from pluripotent cells which are present within scaffold or in the host tissue.^220^ Moreover, BMP-2 and BMP-7 are approved by FDA to be used in treatments of spinal fusions and long-bone fractures in association with a collagen carrier.^221^ The main limitations of the use of BMP-2 are the use of supraphysiological doses which may lead to complications such as immune reactions, formation of ectopic bone tissue, and oedemas.^222,223^ To overcome these limitations, one strategy is to use hydrogels to sequester the growth factors and slowly release them in the site of morbidity for the upregulation of suitable cellular activity. Among the various strategies proposed, there is, for example, the functionalization of hydrogels with heparin because of its affinity for BMP-2.^224,225^ Such systems aim at preventing burst release and favor sustained release of BMP-2 to promote mineral deposition within the injured site.^8^ Thermosensitive hydrogels are also good candidates because they allow to incorporate BMP-2 by simply mixing it at the liquid polymer phase before gelation in situ at physiological temperature. Seo et al. have shown the ability of injectable thermosensitive polymeric nanoparticle hydrogels to efficiently carry and release BMP-2 in a sustained and controlled fashion both in vitro and in vivo. They also showed that in vivo, hydrogel-carried BMP-2 was able to promote new bone generation and infiltration of bone/progenitor cells from the surrounding tissues within the hydrogel without inflammatory responses upon each injection.^221^ Although BMP-2 by itself has always shown strong osteogenic potency, there is still debate about its effects on exogenous grafted cells in tissue engineering products. In fact, BMP-2 remains the most widely studied factor, with a significant action on the survival and differentiation of MSCs;^226^ however, dependently on the tissue of origin of MSC, there are discrepant results concerning the effects of BMP-2. For instance, the efficiency of BMP-2 on ADSCs is controversial, some studies reporting a significant osteogenic effect,^227–229^ and others showing no effect at all.^230^ The action of BMP-2 is more consensual on BMSCs, with positive effects on cell survival in vivo,^220^ and on bone formation. BMP-2 has also been shown to stimulate bone formation by human ESCs^231^ and iPSCs.^192^

As an alternative to BMPs, other growth factors within the above-mentioned list, individually or in combinations, have been shown to have positive effects on MSC survival or MSC-mediated bone formation, such as EGF,^232^ TGF-β and FGF,^233^ and PDGF.^234^ Some bioactive small-molecular-weight compounds have also been shown to favor stem cell–mediated bone formation. For example, some studies report an osteogenic effect of icariin^235^ or simvastatin^236^ loaded inside cellularized BTE scaffolds.

A more recently identified family of molecules that may be used to induce MSC differentiation toward osteoblasts is microRNAs (miRNA).^237^ miRNAs are short single-strand non-coding molecules of RNA (between 18 and 24 nucleotides long) acting in the cytoplasm as gene inhibitors and used by cells to regulate the expression of many genes by RNA interference.^238^ Several gene delivery techniques and approaches can be employed to ferry miRNA to cells, and this approach appears to be a promising tool to stimulate in vivo bone formation in the presence of MSCs.^239,240^ Enhancement of osteogenic or angiogenic properties of MSCs has been achieved by introducing in these cells plasmids or adenoviruses encoding for the expression of growth factors such as BMP-2 or angiopoietin,^241^ respectively. Although these tools have been proved to be usually more efficient than soluble factors^242^ because they allow a continuous, long-term delivery of the active proteins, therapeutic applications are likely to be hindered by risks associated with the introduction of exogenous nucleic acid sequences in grafted cells. Transient transfection of siRNAs may be more acceptable and has been shown to improve MSC performances.^243^

#### Oxygen control

Since stem cells are amplified in 21% O_2_ atmosphere before being grafted into a tissue where O_2_ concentration drops to 3%–5%, a tempting strategy is to pre-condition them so that they can adapt their metabolism to improve their survival and performances once implanted in the harsh tissue environment, at least until neo-vascularization restores normal nutrient and oxygen supply.^244^ Pre-conditioning of MSCs by growing them in hypoxic conditions has shown some benefits on MSC survival and on some of their physiological properties;^245^ but to our knowledge, these strategies have not been explored in the context of in vivo bone formation or bone defect repair. An alternative strategy to help cell surviving the in vivo hypoxic environment is to provide them with an extra oxygen store using synthetic oxygen carriers, which can slowly release oxygen transiently until vascularization is restored.^172,173^ This strategy has been shown to enhance bone formation by implanted MSCs.

#### Mechanical stimulation

Whereas cells are commonly grown in vitro on 2D platforms and under static conditions, a number of studies have shown that growing MSCs in 3D matrices, and in addition under mechanical stimulation, considerably modifies their phenotype as compared to classical 2D culture. Different types of mechanical stimuli can be applied to the cell-containing scaffold. It is possible to apply compressive or tensile loads with defined strength and frequency to the scaffold, resulting in the deformation of its structure and modification of cell adhesion. The effect of compressive loads has been shown to be positive on the in vitro differentiation of MSCs toward osteoblasts,^246^ but its impact on in vivo performances remains unexplored. Another type of stimulation consists of perfusion of the cellularized scaffolds with culture medium in bioreactors, controlling flow rate and pressure. Submitting embedded cells to fluid flow has been shown to enhance their osteoblastic differentiation and bone formation.^247^ Even a short session of fluid perfusion has been shown to increase osteoblast maturation.^248^ Such perfusion systems have also been shown to be effective to produce MSCs, and subsequently osteoblasts, from human ESCs and iPSCs.^249^ These mechanical treatments are, however, difficult to reproduce in all laboratories as the fine control of all the parameters, which considerably influence the effect on cell phenotype, heavily depends on the type of equipment used to generate and control the flow. In addition, their benefit for the in vivo efficiency of MSCs is still not convincingly documented.^250^

### Contribution of exogenous versus host cells in tissue regeneration

Many combinations of scaffold/stem cells/growth factors have proved to be osteogenic and to promote bone defect repair. The benefit of the presence of stem cells within the implanted scaffold has also been demonstrated in a large number of studies. However, one key question is how grafted cells contribute to tissue regeneration. In terms of experimental data, two questions can be formulated: “what percentages of the initially implanted cells are still present within the newly formed tissue at the end of the experiment (when new tissue has been formed), and what is their differentiation status?” and “what is the percentage of exogenous versus host cells which participate in the construction of the new tissue?” These data are usually not available from the published work. In some instances however, careful quantification of human stem cells and host mouse cells has been achieved in the biopsies, and it provides interesting clues. In a recent study, Nuschke et al.^232^ have analyzed the effect of EGF, tethered to tricalcium phosphate particles and embedded in a collagen matrix, together with primary BMSCs. They report a positive effect of EGF on the survival of exogenous cells. But this study also shows that the proportion of exogenous cells is very low (less than 10%), as compared to host cells, and decreases over time. These observations suggest that new tissue is not built primarily by exogenous MSCs, but essentially by endogenous cells. These observations support an indirect, paracrine effect of MSCs. In contrast, some studies provide convincing evidence for the direct involvement of the human grafted cells, supported by the deposition of human collagen.^251^ Two major differences can be noted between both models: in the first study, BMSCs were directly implanted without any pre-culture, whereas in the latter, the construct containing ASCs was incubated for 14 days in an osteogenic medium. Both MSC types were seeded on a β-tricalcium phosphate (TCP) scaffold. Although it is difficult to draw conclusions from this unique comparison, one can speculate that the fate of implanted cells is likely to be affected by the pre-conditioning, in particular submission of the tissue engineering product to a pre-culture or not, and the conditions used for this pre-culture. Another study shows that iPSCs, pre-differentiated into osteoblasts, efficiently promote bone formation and can be quantitatively found after several weeks.^252^ A study by Binder et al. compared MSCs implanted after culture either in basal or in osteogenic medium and observed a very significantly higher survival when cells were pre-cultured in osteogenic medium. In parallel, bone formation is also increased in these conditions.^253^

## Conclusion and future directions

From this review, some conclusions can be drawn but many questions are still pending.

Raising the issue of an ideal “carrier” or “scaffold” for bone repair cells seems at the moment a non-sense, because in fact the definition itself of such a perfect material is not univocal. According to the type of bone defect (anatomical location, size, shape) and to the quality of the surrounding tissues, which depends on the cause of the bone damage and also on several of the patient’s physiological parameters, different materials could be the best choice. Many types of hydrogels have been shown to have osteoinductive and osteogenic properties in the presence of cells and growth factors, but their translation to clinical application relies on other parameters such as injectability, biocompatibility, mechanical stability, and biodegradation rate. These properties may certainly have to be adapted to the specific therapeutic application and, as mentioned above, patient-dependent parameters should be taken into account. Hydrogels appear as the systems of choice for cell transplantation, and many recent studies have pointed out that combinatorial approaches, employing blends of natural and/or synthetic polymers with different properties, are the right way to follow to overcome the limitations of classical hydrogel-forming materials, even if controlling the relevant hydrogel parameters such as mechanical properties, degradability, porosity, biocompatibility, and bioactivity, at the same time, is hardly possible. In addition, the incorporation of calcium phosphate particles, mimicking the inorganic phase of bone ECM, has been very often shown to confer improved osteoconduction and osteoinduction to the scaffold and also osteogenic potential to the grafted cells. One of the greatest challenges in hydrogel-based systems for BTE remains the achievement of suitable mechanical properties for the treatment of load-bearing defects. The possibility of designing scaffolds by 3D printing techniques will certainly enable to better control the structural, and hence mechanical and biological properties of the products. However, the success of these new approaches in bone tissue regeneration largely depends on the capacity of the researchers to model the scaffold they want to produce, and hence to be able to establish clear structure–bioactivity relationships. This is a complex challenge, and whereas so far some success has been met at the in vitro level, the ability to design structures that will be able to fulfill their function once in the complex in vivo environment is still far ahead. Composite systems, combining hydrogels with solid phases (e.g. degradable polymeric structures, bioceramics), are another promising alternative since they can provide synergistic biological activity together with mechanical reinforcement, but their actual in vivo potential still needs to be explored.

If the choice or design of the best scaffold is not easy, choosing the best cell source and the best way to handle and prepare them is another challenging issue. In addition to pure efficiency criteria, the choice of the cell source should also take into account cell availability, costs associated with cell expansion and pre-conditioning, safety issues, and also ethical concerns. Ethical issues are essentially related to the use of ESCs that despite their many practical advantages and their almost unlimited potential cannot be considered at the moment the cell type of choice. iPSCs suffer from difficult handling and insufficient proof of safety, but the rapid development of knowledge and techniques on these cells should rapidly overcome these limitations. Considering all the adult stem-cell sources, there are only minor differences in cell survival, osteoblastic differentiation capacity, and bone forming activity, based solely on the origin of the cells used. These differences can be smoothed by the association with appropriate growth factors, co-embedded with cells and released in a controlled manner by the use of different delivery systems. In conclusion, the choice of the cell source may be governed by practical issues such as availability and costs, and also adapted to the patient’s health status and physiological characteristics. For instance, bone marrow, adipose tissue, and dental pulp represent three major sources of autologous MSCs, and the choice of the donor tissue may be patient-dependent. iPSCs, which also show a high degree of patient donor dependency, should probably be tested for their efficiency before engaging into a long-lasting and probably expensive process. In this context, the identification of markers that would predict the potential of different adult cells from various tissues to generate highly efficient iPSCs could help making the best choice.

If different stem cells are eligible as bone repair cells, very little is known about the mechanism by which they contribute to the bone regeneration process.

In vivo cell survival is still poorly investigated, and very few studies have addressed this question in a quantitative manner. Survival clearly seems favored by pre-culture in an osteogenic medium, and this prolonged lifetime is associated with improved bone formation, suggesting that abundant functional cells are necessary to obtain efficient bone regeneration. The drawback of such procedure is its costs and the risks of genetic alterations upon prolonged culture. The co-grafting of undifferentiated cells combined with the controlled release of osteogenic growth factors is undoubtedly a promising alternative to extensive pre-culture.

A lot of work has yet to be done to characterize the role and the fate of grafted cells. Imaging tools to follow the fate of implanted cells, to localize them and more importantly to quantify them, are available or under rapid development.^254^ The interplay between exogenous and resident cells is another poorly explored question, and the rich secretome of MSCs certainly plays a major role in the recruitment, maturation, and organization of the resident cells within the regenerating tissue. A better knowledge of cell behavior on transplantation will be pivotal in drawing guidelines for the design of hydrogel-based systems with mechanical, structural, and biological properties optimized for osteogenesis.

Finally, the issue of a rapid and efficient vascularization of the grafted TEPs remains one of the most challenging. Endothelial cells or their progenitors and angiogenic growth factors have proved to be efficient in eliciting the formation of new blood vessels. But the anastomosis of these newly formed vessels with those of the host and the quality and function of this neo-vasculature are far from being optimal. In this respect, 3D printing technologies might bring a decisive input, because they allow not only the construction of perfectly controlled scaffold structures (with interconnected pores to allow vessel invasion) but also the printing of endothelial cells progenitors with high resolution, to favor the rapid formation of capillaries within the macroscopic BTE scaffold. Unfortunately, the use of 3D printing is not compatible with the injectability of the system.

To conclude, multicomponent composite systems appear as the new generation of hydrogel-based systems, where incremental improvements obtained in the past research can be merged synergistically. However, it is quite apparent that combining all the desired properties in “ideal” cellularized scaffolds is a utopia and compromises need to be done in their conception. The future solutions to bone repair challenges might come from the application of complementary technologies and techniques based on the precise control, at different scales, of the organization of osteogenic and angiogenic actors in a single, highly structured scaffold where accurately selected and preconditioned cells can find a suitable physiological-like environment to guide bone tissue regeneration.
